# Supplementary figures and images for: Shift of symbiont communities in Acropora tenuis juveniles under heat stress
Source: PeerJ. 2017 Dec 13;5:e4055. doi: 10.7717/peerj.4055 (PMC5732543; doi:10.7717/peerj.4055)

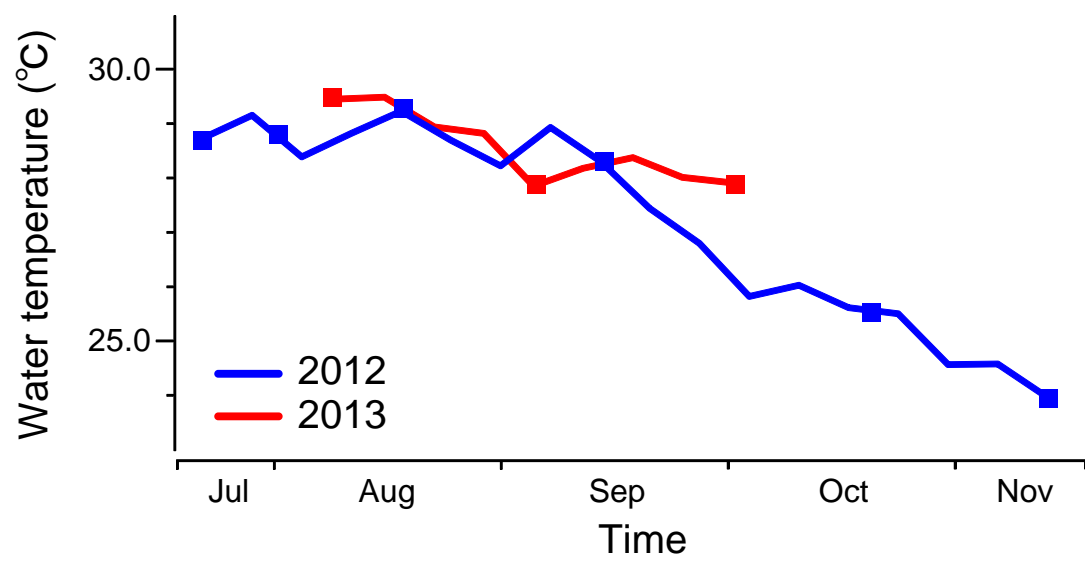

Supplement: Figure S1 — Water temperatures in the ambient experimental tanks. Water temperatures were hourly measured by a logger set in one of the two (in 2012) or three (in 2013) experimental tanks for each temperature treatment. Weekly mean values of the water temperatures in ambient treatment tanks are shown. Symbols marked on the graph indicate when coral juveniles were collected. The average temperature in the ambient treatment tank of the warmest month (August) was 28.74 ±0.71 °C in 2012 and 29.18 ±0.55 °C in 2013. [file peerj-05-4055-s001.pdf]

(A) 2012

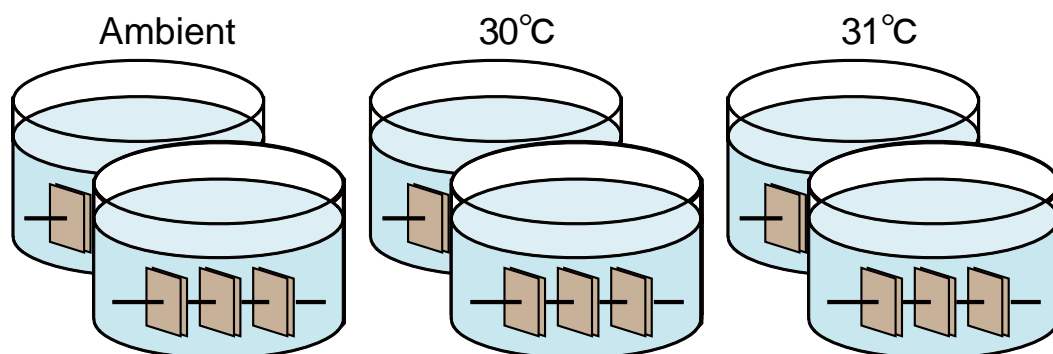

(C)

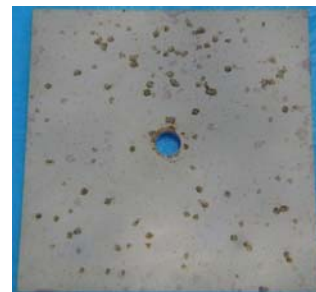

(B) 2013

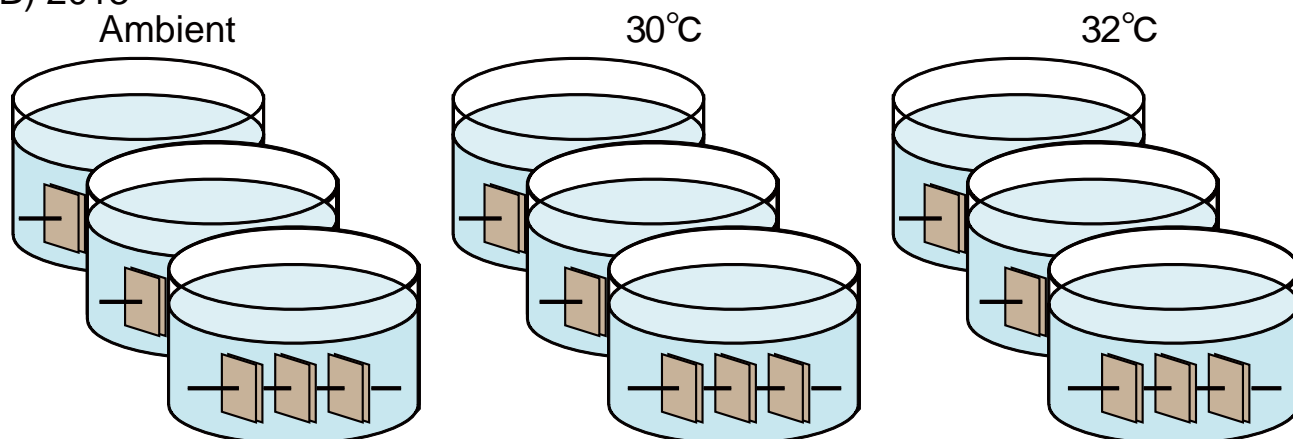

Supplement: Figure S2 — Tank experimental design in 2012 (A) and 2013 (B). (C) is showing a tile (10 cm × 10 cm) at the beginning of the experiment in 2012. [file peerj-05-4055-s002.pdf]

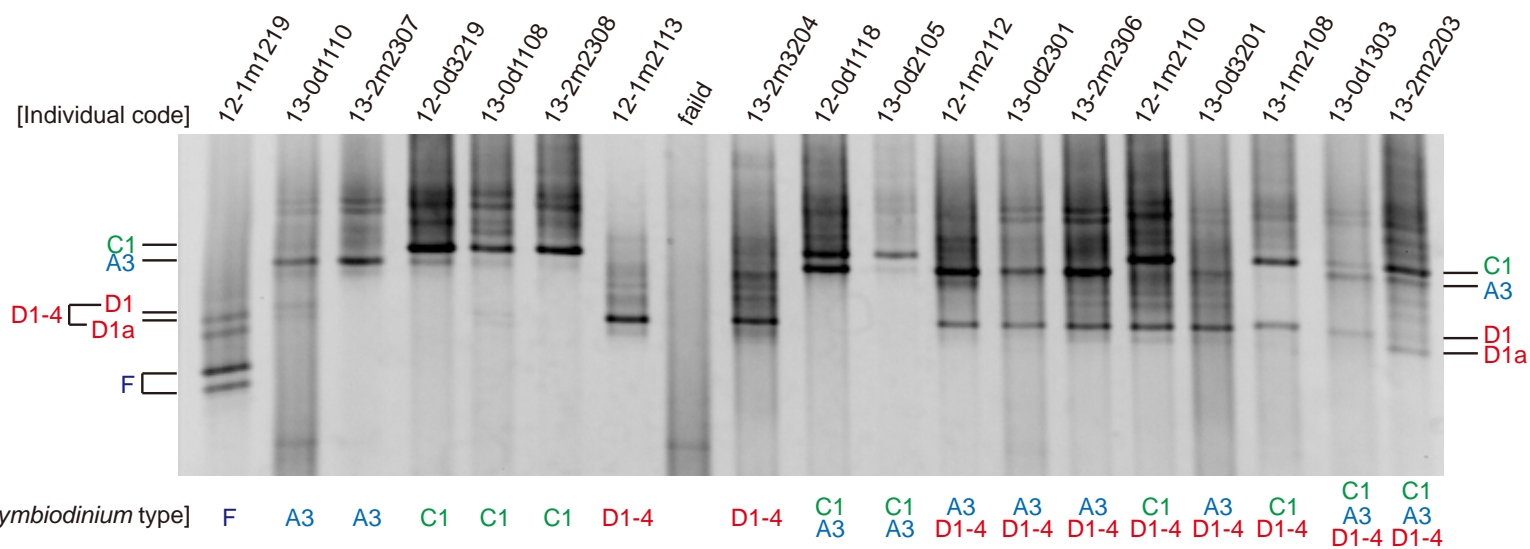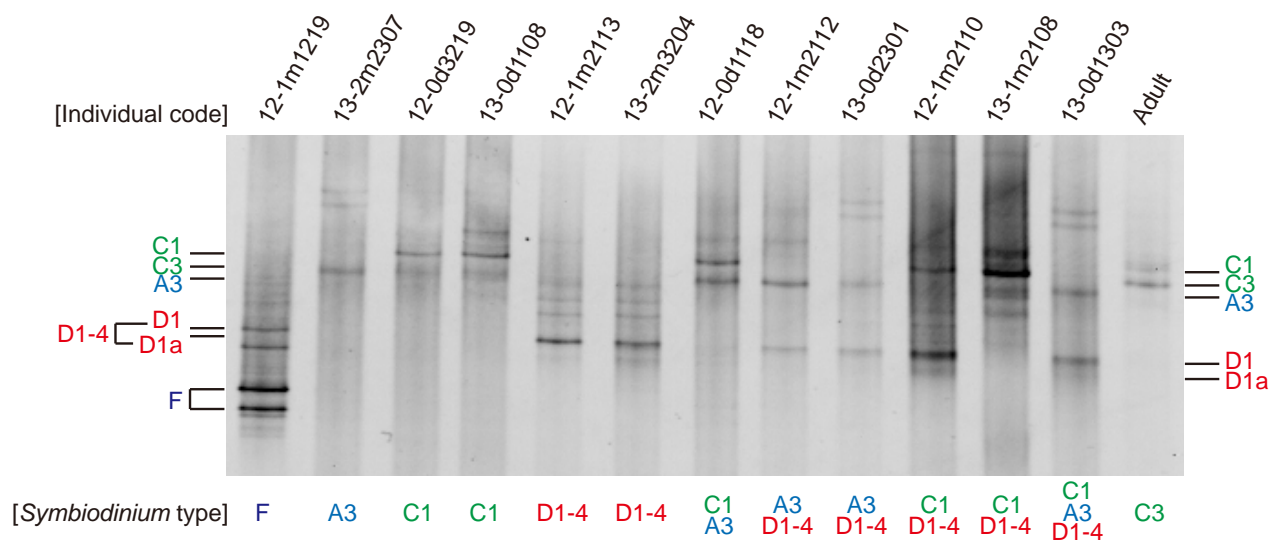

Supplement: Figure S3 — DGGE fingerprints with patterns used to detect Symbiodinium types in this study. Eight patterns of DGGE bands were detected from juvenile corals, while one unique pattern was detected from adult corals. Individual ID indicates [year]-[collection date] [treatment] [replicate] [individual numbers]. See raw data for details of individual coral colonies. [file peerj-05-4055-s003.pdf]

## (A) 2012

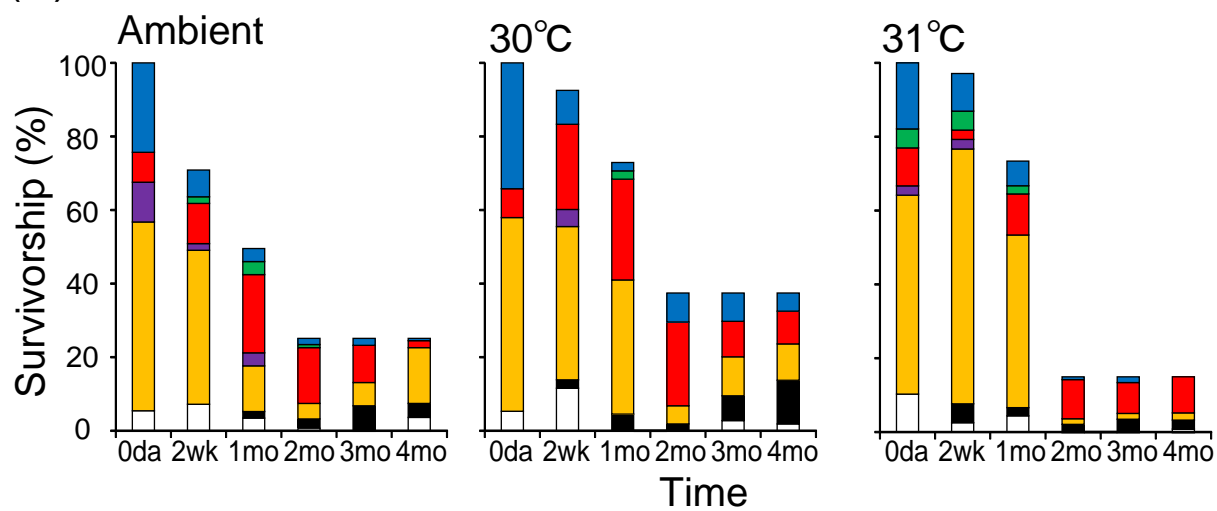

## (B) 2013

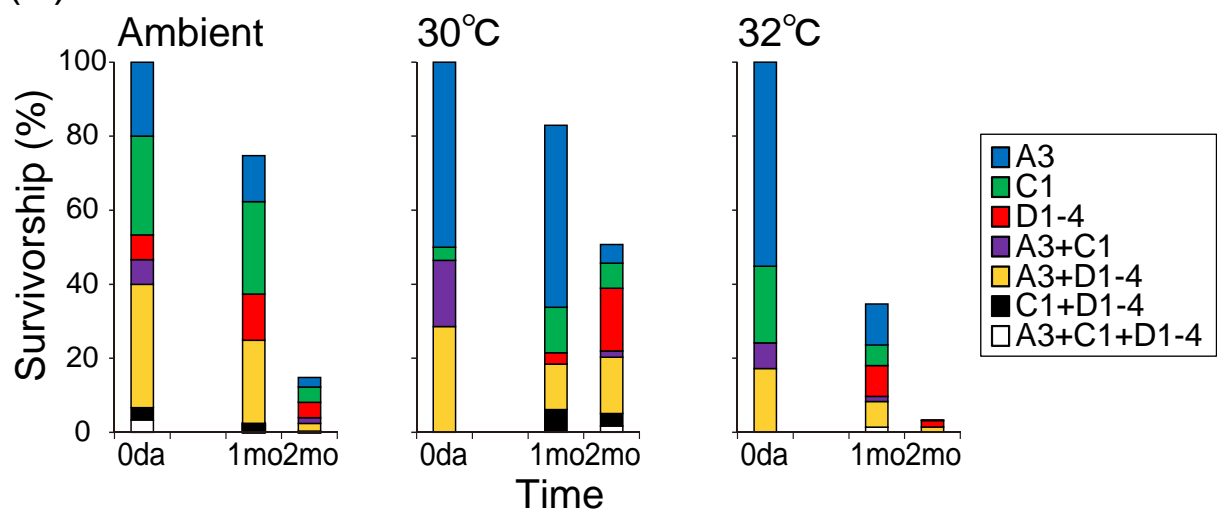

Supplement: Figure S4 — Symbiodinium type compositions within individual colonies of Acropora tenuis juveniles and their occurrences in surviving individuals. Graphs show data combining Figs. 1 and 4. Numbers of individuals analyzed and observed for each composition are shown in Table S2. [file peerj-05-4055-s004.pdf]
