# Supplementary material for: Shift of symbiont communities in Acropora tenuis juveniles under heat stress
Source: PeerJ. 2017 Dec 13;5:e4055. doi: 10.7717/peerj.4055 (PMC5732543; doi:10.7717/peerj.4055)
Supplement: Supplemental Information 1 — Table S1. Numbers and percentages (in parentheses) of colonies infected by each type of Symbiodinium. Table S2. Compositions of Symbiodinium types in each juvenile coral colony and their occurrences (in parentheses are percentages). [file peerj-05-4055-s005.docx]

Table S1 Numbers and percentages (in parentheses) of colonies infected by each type of *Symbiodinium*.

| Year | Treatment | Date | Number of colonies analyzed | *Symbiodinium* type | | | |
| --- | --- | --- | --- | --- | --- | --- | --- |
|  |  |  |  | A3 | C1 | D1-4 | F |
| 2012 | Ambient | 0 da | 37 | 34 (91.9) | 6 (16.2) | 24 (64.9) | - |
|  |  | 2 wk | 39 | 32 (82.1) | 6 (15.4) | 33 (84.6) | - |
|  |  | 1 mo | 29 | 13 (44.8) | 7 (24.1) | 22 (75.9) | 1 (3.4) |
|  |  | 2 mo | 30 | 8 (26.7) | 5 (16.7) | 27 (90.0) | - |
|  |  | 3 mo | 40 | 12 (30.0) | 11 (27.5) | 37 (92.5) | - |
|  |  | 4 mo | 40 | 31 (77.5) | 12 (30.0) | 39 (97.5) | - |
|  |  |  |  |  |  |  |  |
|  | 30 °C | 0 da | 38 | 35 (92.1) | 2 (5.3) | 25 (65.8) | - |
|  |  | 2 wk | 40 | 29 (72.5) | 8 (20.0) | 34 (85.0) | - |
|  |  | 1 mo | 32 | 17 (53.1) | 3 (9.4) | 30 (93.8) | - |
|  |  | 2 mo | 38 | 13 (34.2) | 2 (5.3) | 30 (78.9) | - |
|  |  | 3 mo | 39 | 10 (25.6) | 10 (25.6) | 31 (79.5) | - |
|  |  | 4 mo | 38 | 17 (44.7) | 14 (36.8) | 33 (86.8) | - |
|  |  | 1.5 yr | 12 | - | 3 (25.0) | 11 (91.7) | - |
|  |  |  |  |  |  |  |  |
|  | 31 °C | 0 da | 39 | 33 (84.6) | 7 (17.9) | 29 (74.4) | - |
|  |  | 2 wk | 38 | 33 (86.8) | 6 (15.8) | 31 (81.6) | - |
|  |  | 1 mo | 33 | 26 (78.8) | 4 (12.1) | 29 (87.9) | - |
|  |  | 2 mo | 20 | 3 (15.0) | 3 (15.0) | 19 (95.0) | - |
|  |  | 3 mo | 29 | 6 (20.7) | 7 (24.1) | 26 (89.7) | - |
|  |  | 4 mo | 31 | 6 (19.4) | 7 (22.6) | 31 (100.0) | - |
|  |  |  |  |  |  |  |  |
| 2013 | Ambient | 0 da | 30 | 19 (63.3) | 12 (40.0) | 14 (46.7) | - |
|  |  | 1 mo | 30 | 14 (46.7) | 11 (36.7) | 15 (50.0) | - |
|  |  | 2 mo | 29 | 12 (41.4) | 12 (41.4) | 13 (44.8) | - |
|  |  |  |  |  |  |  |  |
|  | 30 °C | 0 da | 28 | 27 (96.4) | 6 (21.4) | 8 (28.6) | - |
|  |  | 1 mo | 27 | 20 (74.1) | 6 (22.2) | 7 (25.9) | - |
|  |  | 2 mo | 30 | 14 (46.7) | 8 (26.7) | 22 (73.3) | - |
|  |  |  |  |  |  |  |  |
|  | 32 °C | 0 da | 29 | 23 (79.3) | 8 (27.6) | 5 (17.2) | - |
|  |  | 1 mo | 25 | 15 (60.0) | 6 (24.0) | 12 (48.0) | - |
|  |  | 2 mo | 21 | 9 (42.9) | 1 (4.8) | 20 (95.2) | - |

Table S2 Compositions of *Symbiodinium* types in each juvenile coral colony and their occurrences (in parentheses are percentages).

| Year | Treatment | Date | Number of colonies analyzed | *Symbiodinium* types | | | | | | | |
| --- | --- | --- | --- | --- | --- | --- | --- | --- | --- | --- | --- |
|  |  |  |  | A3 |  |  | A3 | A3 |  | A3 |  |
|  |  |  |  |  | C1 |  | C1 |  | C1 | C1 |  |
|  |  |  |  |  |  | D1-4 |  | D1-4 | D1-4 | D1-4 |  |
|  |  |  |  |  |  |  |  |  |  |  | F |
| 2012 | Ambient | 0 da | 37 | 9 (24.3) | - | 3 (8.1) | 4 (10.8) | 19 (51.4) | - | 2 (5.4) | - |
|  |  | 2 wk | 39 | 4 (10.3) | 1 (2.6) | 6 (15.4) | 1 (2.6) | 23 (59.0) | - | 4 (10.3) | - |
|  |  | 1 mo | 29 | 2 (6.9) | 2 (6.9) | 12 (41.4) | 2 (6.9) | 7 (24.1) | 1 (3.4) | 2 (6.9) | 1 (3.4) |
|  |  | 2 mo | 30 | 2 (6.7) | 1 (3.3) | 18 (60.0) | - | 5 (16.7) | 3 (10.0) | 1 (3.3) | - |
|  |  | 3 mo | 40 | 3 (7.5) | - | 16 (40.0) | - | 10 (25.0) | 11 (27.5) | - | - |
|  |  | 4 mo | 40 | 1 (2.5) | - | 3 (7.5) | - | 24 (60.0) | 6 (15.0) | 6 (15.0) | - |
|  |  |  |  |  |  |  |  |  |  |  |  |
|  | 30 °C | 0 da | 38 | 13 (34.2) | - | 3 (7.9) | - | 20 (52.6) | - | 2 (5.3) | - |
|  |  | 2 wk | 40 | 4 (10.0) | - | 10 (25.0) | 2 (5.0) | 18 (45.0) | 1 (2.5) | 5 (12.5) | - |
|  |  | 1 mo | 32 | 1 (3.1) | 1 (3.1) | 12 (37.5) | - | 16 (50.0) | 2 (6.3) | - | - |
|  |  | 2 mo | 38 | 8 (21.1) | - | 23 (60.5) | - | 5 (13.2) | 2 (5.3) | - | - |
|  |  | 3 mo | 39 | 8 (20.5) | - | 10 (25.6) | - | 11 (28.2) | 7 (17.9) | 3 (7.7) | - |
|  |  | 4 mo | 38 | 5 (13.2) | - | 9 (23.7) | - | 10 (26.3) | 12 (31.6) | 2 (5.3) | - |
|  |  | 1.5 yr | 12 | - | 1 (8.3) | 9 (75.0) | - | - | 2 (16.7) | - | - |
|  |  |  |  |  |  |  |  |  |  |  |  |
|  | 31 °C | 0 da | 39 | 7 (17.9) | 2 (5.1) | 4 (10.3) | 1 (2.6) | 21 (53.8) | - | 4 (10.3) | - |
|  |  | 2 wk | 38 | 4 (10.5) | 2 (5.3) | 1 (2.6) | 1 (2.6) | 27 (71.1) | 2 (5.3) | 1 (2.6) | - |
|  |  | 1 mo | 33 | 3 (9.1) | 1 (3.0) | 5 (15.2) | - | 21 (63.6) | 1 (3.0) | 2 (6.1) | - |
|  |  | 2 mo | 20 | 1 (5.0) | - | 14 (70.0) | - | 2 (10.0) | 3 (15.0) | - | - |
|  |  | 3 mo | 29 | 3 (10.3) | - | 16 (55.2) | - | 3 (10.3) | 7 (24.1) | - | - |
|  |  | 4 mo | 31 | - | - | 20 (64.5) | - | 4 (12.9) | 5 (16.1) | 2 (6.9) | - |
|  |  |  |  |  |  |  |  |  |  |  |  |
| 2013 | Ambient | 0 da | 30 | 6 (20.0) | 8 (26.7) | 2 (6.7) | 2 (6.7) | 10 (33.3) | 1 (3.3) | 1 (3.3) | - |
|  |  | 1 mo | 30 | 5 (16.7) | 10 (33.3) | 5 (16.7) | - | 9 (30.0) | 1 (3.3) | - | - |
|  |  | 2 mo | 29 | 5 (17.2) | 8 (27.6) | 8 (27.6) | 3 (10.3) | 4 (13.8) | 1 (3.4) | - | - |
|  |  |  |  |  |  |  |  |  |  |  |  |
|  | 30 °C | 0 da | 28 | 14 (50.0) | 1 (3.6) | - | 5 (17.9) | 8 (28.6) | - | - | - |
|  |  | 1 mo | 27 | 16 (59.3) | 4 (14.8) | 1 (3.7) | - | 4 (14.8) | 2 (7.4) | - | - |
|  |  | 2 mo | 30 | 3 (10.0) | 4 (13.3) | 10 (33.3) | 1 (3.3) | 9 (30.0) | 2 (6.7) | 1 (3.3) | - |
|  |  |  |  |  |  |  |  |  |  |  |  |
|  | 32 °C | 0 da | 29 | 16 (55.2) | 6 (20.7) | - | 2 (6.9) | 5 (17.2) | - | - | - |
|  |  | 1 mo | 25 | 8 (32.0) | 4 (16.0) | 6 (24.0) | 1 (4.0) | 5 (20.0) | - | 1 (4.0) | - |
|  |  | 2 mo | 21 | - | 1 (4.8) | 11 (52.4) | - | 9 (42.9) | - | - | - |
